# Medical News and Miscellany

**Published:** 1897-02

**Authors:** 


### Medical News and Miscellany.

**Dr. J. A. Abney** has removed from Lampasas to Brownwood, Texas.

**Dr. C. McGarrity** has removed from Kyle to Leesville, Gonzales county.

**Dr. David Cerna,** of the Texas Medical College, has been appointed Mexican Consul at Galveston.

**Dr. J. D. Westervelt, Jr.,** has removed from Alice, Texas, to Dallas, where he will permanently reside.

**Errata.**—In our January number, the name of Prof. Smith appeared as "Albert J. Smith." It should have been "Allen J. Smith." On page 404, first word of third line from the bottom, for "fort" read "*forte.*"

**Dr. E. E. Scott,** of Demings' Bridge, is taking a special course at the Kentucky School of Medicine at Louisville.

---

**The** JOURNAL calls attention to the professional card of Proferror Emory Lanphear, M. D., of St. Louis, the eminent Surgeon Gynecologist.

---

**Dr. H. W. Cummings,** of Hearne, is taking a course at the New York Polyclinic, and wants the "Red Back" sent to him there until further notice.

---

**A "Peculiarity" not Confined to Doctors.**—Under the head of "Peculiarities of Some Medical Journalists," the *American Medical Journal* (Dr. Chas. Wood Fassett) says: "Dr. Bransford Lewis was married recently," etc. Is getting married one of Dr. Lewis "peculiarities?" Reminds us of a wedding we once saw announced under the head of "Amusements."

---

**The Sultan Drug Co.,** St. Louis, has been granted by the U. S. Circuit Court (Northern District of Illinois) a decree against G. P. Engelhard & Co., of Chicago, enjoining them from publishing or circulating any formula for making "Cactina Pillets" or "Seng." Geo. P. Engelhard & Co. have been publishing what purported to be working formulas for Cactina Pillets and Seng, two preparations manufactured solely by the Sultan Drug Co.; the court thus protecting their trade-rights.

---

**Kind Words.**—The following, from the wide-awake, able and plucky *Atlanta Medical and Surgical Journal*, makes us feel proud: (but we still speak to plain people.) That's what's the matter: The *Red Back* is a free-lance; its policy is "see (a)head and hit it,"—(if it is a quack's head); and it *is* the uncompromising foe and never-let-up antagonist of everything like shenanegan in medicine:

"A JOURNAL that gladdens the heart and delights the understanding every month is the *Texas Medical*, otherwise known as the "*Red Back*." As Chimmie Fadden would say, *it is up to de limit*. Standing for the ethical in both medicine and journalism, it is the uncompromising foe to the medical pretender and adventurer. It is a joy to its friends and a terror to its enemies; *suaviter in modo*, *fortiter in re*. There is but one *Texas Medical Journal*, and Brother Daniel is the editor thereof. May its vigor never perish, nor its complexion fade."

Yea, yea: he that taketh not the *Red Back* getteth left. See?

**In the** Texas legislature, Dr. J. S. Morris, senator from Cass county, is chairman of the committee on Public Health, and Dr. Yett, senator from Burnet and Travis, is a member of the same committee. In the house, are Drs. A. C. Oliver, representative from Cass, Flint from Marion, L. D. Hill from Travis, Field from Grayson. These are all the doctors in the legislature, we believe—six in all. It is to be hoped these gentlemen will be strong allies with the profession in the effort to secure medical legislation.

---

**Dr. R. H. L. Bibb,** of Mexico City, who already holds the position of Chief surgeon of the Mexican National Railway Company, has been complimented by the election by the board of managers of the American Hospital in the City of Mexico to the responsible position of physician and surgeon to that hospital.

This is the highest preferment in the gift of the Americans living in Mexico. It shows the esteem in which the doctor is held by his fellow countrymen in our sister republic, and the choice does credit to their good sense and powers of discrimination.

---

"**The Magazine of Medicine,** formerly *Moody's Magazine of Medicine*, is a unique medico-literary magazine, illustrated, published monthly by the Magazine of Medicine Co., Atlanta, Ga. Dr. Ralcy H. Bell, editor-in-chief, has sole charge or supervision of the business and all other interests of the publication." Subscription price $2.00 per year.

The above is the announcement at the head of the editorial page of Vol. 2, No. 1, of the successor to the nondescript *Moody's Medical Magazine*. Dr. Bell is to be congratulated upon the change, and upon issuing a very handsome, interesting and attractive magazine.

---

**Death of Dr. Gregg.**—Dr. Richard S. Gregg, of Manor, Texas, died at his home in Manor, on the 22nd of January (ult.) after a brief illness. Dr. Gregg was a graduate of the Medical Department of Tulane University, of New Orleans, of the Class of 1873. He was in the prime of life; not over 48 years of age, and was apparently in the best of health. His death was quite unexpected, and a great shock to his friends. Dr. Gregg was a good and useful man, and his death is a serious loss to the medical profession and to the public. He was a member of the State Medical Association, and of the Austin District Medical Society, and took an active interest in medical matters.

**Death of Mr. Von Boeckmann.**—In common with this community, the JOURNAL has sustained a great loss in the death of Eugene Von Boeckmann. He died on the 28th of January, ult., after a brief illness, and his death was a great shock to the community. He was the JOURNAL's printer, and printed the first edition, nearly twelve years ago; he was a factor in its growth and developement. The JOURNAL is in mourning to-day, truly; for in the death of Mr. Von Boeckmann we feel that we have sustained a personal loss. He was the sole owner of Austin's great printing and bookbinding establishment, and had built it up from nothing by his labor and sagacity. Mr. Von Boeckmann was an excellent man in every relation of life, a model husband and father, a strong friend, a public spirited citizen, and a generous benefactor to the poor. His loss will be felt by all, but most by those who knew him best, for only those who knew him well could appreciate the true nobleness of his nature. The JOURNAL extends its condolence to his bereaved family, and mingles its regrets with those of thousands of attached friends.

